# On the Use of Opium

**Published:** 1860-03

**Authors:** Charles Brackett

**Affiliations:** Rochester, Fulton Co., Ind.


### ON THE USES OF OPIUM.

BY CHARLES BRACKETT, M. D., OF ROCHESTER, FULTON CO., IND.

*Editors of the Journal:*—

I have read with pleasure and profit, the article on opium, by Dr. Hudson, in the January number of the *Journal* of this year.

Its effects, in some varieties of chronic ulcers, are often most astonishing. My attention was drawn to its use for the cure of indolent ulcers, by an article in some one of the *Journals* several years ago. I do not recollect now what *Journal*, but it has always since done all that is claimed for it in my hands; and I could give up all other medicines for these common and stubborn affections except opium. I usually direct a full dose, sufficient to produce free diaphoresis at bed time.

My experience with it in Typhoid fever is very limited as the disease seldom occurs in my practice.

For inflammatory diseases generally, and especially those of the mucous membranes, full diaphoretic doses in the commencement of the disease, and repeated as occasion requires, are frequently all-sufficient to cut short the disease within twenty-four or forty-eight hours.

I was first led to its use in catarrh, or cold in the head, by an article in one of the first numbers of the *London Lancet* that I ever saw. I was then a student with Dr. Mungo White, of Cherry Valley, N. Y. Subject then to the disease myself, I took ten grain doses of Dover powder, every twenty minutes, till free diaphoresis came on. The next morning, to my surprise, I was cured. The mucous membrane of the nose and the conjunctiva, instead of secreting a thin, acrid fluid excoriating cheeks and lips, felt well and free from all discomfort, and a distressing affection, usually lasting about a week, was cut short in one night. I next tried the remedy on a niece of the Doctor's, who was also habitually subject to catarrh. I showed her the article in the Journal, told her how much I was benefitted by it. She was anxious to try it; and upon her promising not to tell the Doctor that I had so commenced practice, I gave her the Dover powders in ten grain doses, till sweating supervened. A complete cure was the result; she getting up next moruing free from all symptoms of cold in the head. Since that time, now probably seventeen or eighteen years, I have never known it to fail in a single instance, when it could be taken as above. I have, however, for the past seven or eight years, almost discarded the Dover powder in *toto*, using the crude opium, or by times morphine alone.

The following is a case of croup, completely cured by a large dose of morphine:—

Anna Brackett, my daughter, æt. about fourteen months, had for several nights in succession croupal symptoms, with raging cough, feverish action, and considerable difficulty in breathing during the day. I had been absent, and she had taken only household remedies. On the evening of my return home, her symptoms were such as made me quite uneasy. I mixed about one grain of Sul. Morphiæ in about ten teaspoonsful of onion juice well sweetened with honey, and directed a teaspoonful every half hour. I gave the first dose; my wife not understanding fully the directions, and supposing the medicine in the saucer was to be given *ad libitum*, gave the child what it would drink. It soon becoming so quiet, my wife was frightened, and called me to see what was wrong. I asked about the medicine, and she told me what she had done. I did not tell her that I was uneasy, or that she had done wrong, but with my fingers on its little wrist, and sleepless eyes, for some time watched carefully for symptoms of dangerous portent; but they did not come; on the contrary, quiet sleep, easy breathing, and free perspiration followed this dose of over *half a grain of morphine* to a child but a trifle over a year old. On the next morning, bright eyes, a cheery laugh, perfect ease of respiration, and freedom from fever, told the tale of recovery; and no symptom of croup has ever since attacked our youngest born.

I have treated many cases of dysentery with morphine or opium alone; except the using of free injections of warm water, milk, or starch, to wash the mucous coat of the rectum of irritant secretions; and with such treatment am better satisfied than with the more complex and torturing modes.

I look upon opium as one of the most perfect antiphlogistic remedies we possess. It will deplete the system of quarts by the skin, when ounces taken from the vein would leave the system more prostrate and less comfortable. By this free cutaneous drain, we probably rid the system of morbid matters or humors, if any such are present, while by the lancet we deprive the system of so much life. "The blood thereof is the life thereof." The superiority of opium over the lancet, is best shown in pleurisy, where diaphoresis induced by opium makes the more perfect and speedy cure.

I came to this country in the fall of 1845; during the winter following, we had many cases of pneumonia. Within a short period, my brothers (James and Lyman, then practicing here) sent me to see four cases which proved fatal within a few hours from the time I saw them. They had been treated by other practitioners according to the old rule of puking, purging, and bleeding; after seeing the fourth case die, and on my return to the office, I threw the pill-bags under the counter, and remarked that if there were any more patients to visit in *articulo mortis*, they might count me out, for I would not see another second hand case for a short time at least. They both laughed heartily at me, and after I cooled down, they told me they had sent me to see those cases, for the sake of teaching me a lesson in the treatment of pneumonia; that they knowing how these patients had been treated, knew what the finale would be; that I must depend less on the lancet; more on opium, quinine, blisters and antimony; being careful not to use cathartics; and that especially I must depend more on quinine and opium, with supporting adjuvants, for the cure of pneumonia attacking those long resident in this miasmatic region. Since that time, fifteen years, I do not think I have seen four fatal cases of pneumonia; and during the whole time I have been in active practice, except during the winter of 1848–9.

That this result is attributable in a great measure to the lessons I received from my brothers in the fall and early winter of 1845, I have no doubt; if I am successful at all in the treatment of pneumonia, opium is one of, or rather the head agent.

